# Dysregulation of Microglial Function Contributes to Neuronal Impairment in Mcoln1a-Deficient Zebrafish

**DOI:** 10.1016/j.isci.2019.02.031

**Published:** 2019-03-02

**Authors:** Wan Jin, Yimei Dai, Funing Li, Lu Zhu, Zhibin Huang, Wei Liu, Jianchao Li, Mingjie Zhang, Jiulin Du, Wenqing Zhang, Zilong Wen

**Affiliations:** 1 Division of Life Science, State Key Laboratory of Molecular Neuroscience and Center of Systems Biology and Human Health, the Hong Kong University of Science and Technology, Clear Water Bay, Kowloon, Hong Kong, PR. China; 2 Institute of Neuroscience, State Key Laboratory of Neuroscience, CAS Center for Excellence in Brain Science and Intelligence Technology, Chinese Academy of Sciences, 320 Yue-Yang Road, Shanghai 200031, PR. China; 3 Department of Cell, Developmental and Integrative Biology, School of Medicine, South China University of Technology, Guangzhou 510006, PR. China; 4 Shenzhen Key Laboratory for Neuronal Structural Biology, Biomedical Research Institute, Shenzhen Peking University−Hong Kong University of Science and Technology Medical Center, Shenzhen 518036, PR. China

**Keywords:** Cellular Neuroscience, Clinical Neuroscience, Model Organism, Neuroscience

## Abstract

Type IV mucolipidosis (ML-IV) is a neurodegenerative lysosome storage disorder caused by mutations in the *MCOLN1* gene. However, the cellular and molecular bases underlying the neuronal phenotypes of ML-IV disease remain elusive. Using a forward genetic screening, we identified a zebrafish mutant, *biluo*, that harbors a hypomorphic mutation in *mcoln1a*, one of the two zebrafish homologs of mammalian *MCOLN1*. The *mcoln1a*-deficient mutants display phenotypes partially recapitulating the key features of ML-IV disorder, including the accumulation of enlarged late endosomes in microglia and aberrant neuronal activities in both spontaneous and visual-evoking conditions in optic tectal neurons. We further show that the accumulation of enlarged late endosomes in microglia is caused by the impairment of late endosome and lysosome fusion and the aberrant neuronal activities can be partially rescued by the reconstitution of Mcoln1a function in microglia. Our findings suggest that dysregulation of microglial function may contribute to the development and progression of ML-IV disease.

## Highlights

• *mcoln1a*-deficient fish display microglia impairment and aberrant neuronal activity
• The aberrant neuronal activity can be rescued by expressing WT *mcoln1a* in microglia
• Impairment of microglia-neuron contact contributes to the aberrant neuronal activity

## Graphical Abstract

**Figure:**
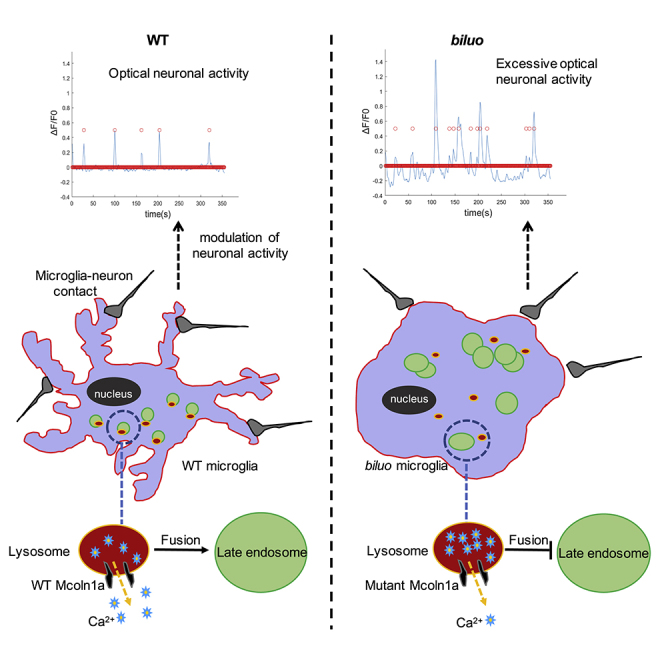

## Introduction

Microglia are the resident macrophages in the central nervous system (CNS) (Hanisch and Kettenmann, 2007, Kim and de Vellis, 2005). They were initially characterized by Spanish neuroscientist del Rio Hortega in 1919 as a population of cells in the brain distinct from neurons, oligodendrocytes, and astrocytes (Hortega, 1919). For years, it has been thought that microglia predominately act as immune cells to remove cellular debris and infectious agents in the CNS (Kreutzberg, 1995, Napoli and Neumann, 2009). But recent studies have revealed that, in addition to functioning as immune response, microglia also play critical roles in the regulation of neural development and functions (Bialas and Stevens, 2013, Li et al., 2012b, Paolicelli et al., 2011, Schafer et al., 2012, Squarzoni et al., 2014, Tremblay et al., 2010, Wake et al., 2009). More importantly, aberrant microglia activities have been found to be closely associated with a number of neurodegenerative disorders, including Multiple Sclerosis, Autism, and Alzheimer and Parkinson diseases (Chastain et al., 2011, Davoust et al., 2008, Derecki et al., 2012, Graeber and Streit, 2010, Iaccarino et al., 2016, Kim and de Vellis, 2005, Ponomarev et al., 2005). Although the precise underlying mechanism remains unclear, it is generally believed that pathological stimuli such as amyloid β plaques can trigger the activation of microglia to release pro-inflammatory and neurotoxic factors, which can cause neuronal cell death and neural circuit impairment (Block et al., 2007, Kim et al., 2015, Qin et al., 2004, Takeuchi et al., 2006, Thompson and Tsirka, 2017). On the other hand, several studies have indicated that microglia play a beneficial role during the onset and the progression of Alzheimer disease by removing the amyloid β plaques accumulated in the CNS (Herz et al., 2017, Hickman et al., 2008). Thus, a comprehensive understanding of the precise roles of microglia in different neurodegenerative disorders may provide therapeutic treatment for these disorders.

Type IV mucolipidosis (ML-IV) is a neurodegenerative and lysosomal storage disorder with various neuronal symptoms, including psychomotor retardation, hypotonia, corneal opacities, and retinal degeneration (Bargal et al., 2000, Bassi et al., 2000, Wakabayashi et al., 2011). Patients with severe ML-IV display failure of sitting, crawling, and standing within infancy as well as are non-verbal and blind during the teenage stage (Folkerth et al., 1995). Genetically, ML-IV disorder is caused by mutations in transient receptor potential cation channel, mucolipin 1 (MCOLN1), a cation-permeable channel predominantly expressing on the membrane of late endosomes and lysosomes (LELs) (Bach, 2005, Manzoni et al., 2004). It is known that MCOLN1 channel releasing ions from the lumen of LELs to cytosol in response to different stimuli and the MCOLN1-mediated Ca^2+^ efflux are critical for endosomal acidification and organelle homeostasis (Dong et al., 2008, Dong et al., 2009, Dong et al., 2010, LaPlante et al., 2002, LaPlante et al., 2004, Luzio et al., 2007, Morgan et al., 2011). Studies in animal models have revealed that excessive neuronal cell death and muscle dystrophy contribute to the development of ML-IV disorder (Li et al., 2017, Venkatachalam et al., 2008). Intriguingly, histological staining of human brains of patients with ML-IV has revealed that both neurons and microglia display abnormal morphology such as the accumulation of membrane-bounded storage bodies in cytoplasm (Folkerth et al., 1995), indicating that both neurons and microglia contribute to the development of the disease. This notion is further supported by the studies in *MCOLN1*-deficient mice and drosophila, showing that transplantation of wild-type (WT) bone marrow cells into *MCOLN1*-deficient mice and reconstitution of WT *MCOLN1* in hemocytes in *mcoln1*-deficient drosophila can rescue, at least in part, the motor deficiency (Walker and Montell, 2016, Venkatachalam et al., 2008). However, since drosophila lacks microglia and bone marrow cell transplantation in mice has a relatively low efficiency of microglia reconstitution, whether microglia contribute to the development of ML-IV disorder and the underlying mechanism remain to be clarified.

Zebrafish has recently emerged as an alternative vertebrate model system for the study of microglia development and homeostasis (Casano and Peri, 2015). Because of its small size and transparency, zebrafish provides a platform to perform unbiased large-scale forward genetic screening and *in vivo* time-lapse imaging analysis (Driever et al., 1996, Haffter et al., 1996). The development and function of microglia appear to be evolutionarily conserved between zebrafish and mammals (Casano and Peri, 2015). In zebrafish, microglia begin to populate the brain from 2.5 days post fertilization (dpf) (Herbomel et al., 1999, Herbomel et al., 2001). This early population of microglial precursors originates from the rostral blood island (Xu et al., 2015), and they are promoted to colonize the developing zebrafish brain by the signals released from the apoptotic neurons, which occurs naturally during neurogenesis (Xu et al., 2016, Casano et al., 2016) and by the Il34-Csf1r signaling pathway (Wu et al., 2018). Similar to mammalian microglia, zebrafish microglia are capable of removing cellular debris in the CNS and are actively involved in neural activity (Li et al., 2012b, Peri and Nusslein-Volhard, 2008). A recent study has shown that zebrafish consists of two orthologs, *mcoln1a* and *mcoln1b*, of *MCOLN1*, and *mcoln1ab* double-mutant zebrafish display retinal and neuromuscular defects similar to the patients with ML-IV with *MCOLN1* deficiency (Folkerth et al., 1995, Li et al., 2017, Wakabayashi et al., 2011). Thus, zebrafish appears to be a suitable model for studying the role of microglia in the pathogenesis of ML-IV disorder.

Here, we report the isolation and characterization of a zebrafish mutant *biluo*, which harbors a hypomorphic mutation in the *mcoln1a* gene, one of the two zebrafish homologs of *MCOLN1*. We found that loss of Mcoln1a function blocks late endosome and lysosome fusion in microglia and neurons. Moreover, mcoln1a-deficient zebrafish exhibit aberrant neuronal activities at early embryonic stages, which is caused at least in part by the dysregulation of microglial function.

## Results

### *biluo* Mutant Microglia Display Aberrant Morphologies at Embryonic Stage

**Figure 1:**
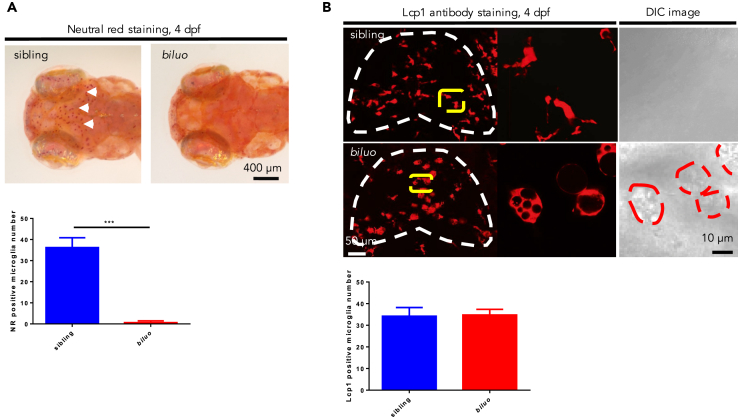
Aberrant Morphology of Embryonic Microglia in *biluo* Mutants (A) Neutral red staining of 4-dpf siblings and mutants. Abundant microglia (white arrows) are positive for neutral red in the siblings (left) but not in the mutants. Quantification data of neutral red positive microglia number in 4-dpf siblings and mutants. n(sibling) = 7 embryos, n(*biluo*) = 6 embryos. ***p < 0.001 Error bars represent mean ± SD. (B) Lymphocyte cytosolic lplastin1 (Lcp1) antibody staining and DIC images of the brains of 4 dpf embryos indicate that microglia are present in *biluo* mutants but exhibit an abnormal enlarged morphology with accumulation of vacuoles. Quantification data of Lcp1 positive microglia number in 4 dpf siblings and mutants. White dashed lines indicate the optic tectum, yellow lines indicate the microglia presented in high magnification view on the right, and red dashed lines indicate the mutant microglia under DIC view. n(sibling)= 4 embryos, n(*biluo*)=6 embryos. Error bars represent mean ± SD.

To uncover regulators involved in the development and function of microglia, we carried out an N-ethyl-N-nitrosourea-based forward genetic screening in zebrafish to search for mutants defective in microglia (Venugopal et al., 2007). Neutral red, a classical histological dye that can accumulate in lysosomes (Herbomel et al., 2001), was used as a marker to track microglia in zebrafish embryos. One mutant, *biluo*, which lacked neutral red staining in the brain but maintained normal general development (Figure 1A), was identified and selected for further in-depth study.

To further characterize the microglia phenotype in *biluo* mutants, we examined the expression of other microglia markers, including lymphocyte cytosolic *plastin 1* (*lcp1*), a myeloid marker for macrophages and their precursors (Herbomel et al., 1999, Jin et al., 2012), in *biluo* mutants. As shown in Figure 1B, anti-Lcp1 antibody staining revealed a comparable number of Lcp1^+^ microglia in the brains of *biluo* mutants and siblings (Figure 1B), indicating that the lack of neutral red staining in *biluo* mutant fish is not due to the absence of microglia but rather attributed to the functional impairment of these cells. This notion was further supported by the differential interference contrast (DIC) microscopic analysis, which showed that *biluo* mutant microglia displayed an enlarged and round-shaped morphology with the accumulation of membrane-bound storage bodies in the cytoplasm (Figure 1B).

### The *biluo* Gene Encodes Mcoln1a Protein

**Figure 2:**
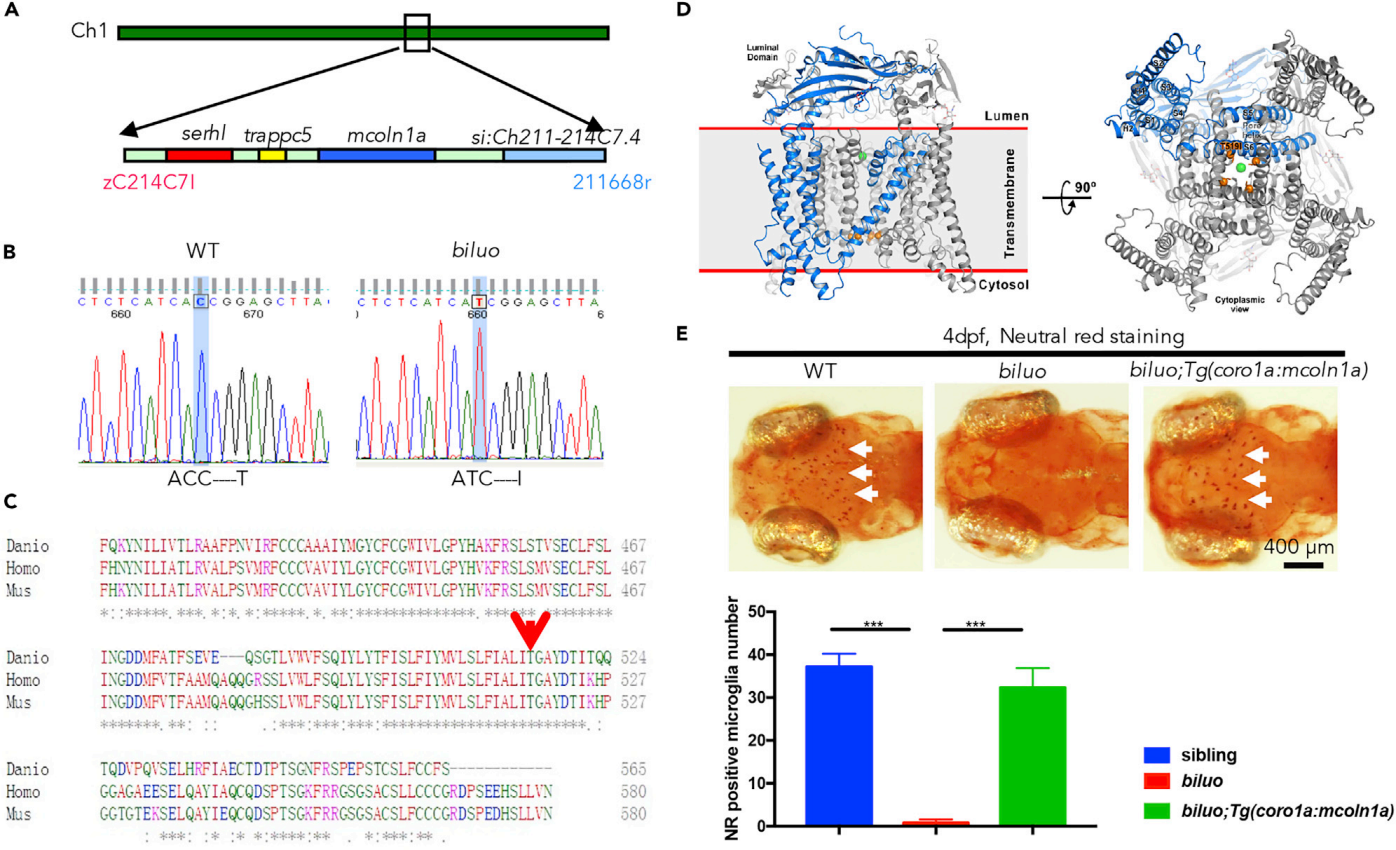
The Microglia Defect in *biluo* Mutants Is Caused by a Point Mutation in *mcoln1a* (A) Positional cloning maps the *biluo* mutation to an 82-kb region on chromosome 1 between two SSLP markers, zC214C7I and 211668r. This region contains four genes: *serhl*, *trappc5*, *mcoln1a*, and *si*:*Ch211-214C7*.*4*. (B) Sequencing the coding regions of the four candidate genes revealed a C to T point mutation in the *mcoln1a* gene, which leads to the change of amino acid T519 to (I). (C) Alignment of human (Homo) MCOLN1, mouse (Mus) MCOLN1, and zebrafish (Danio) Mcoln1a protein sequence. Red arrow indicates the conserved 519^th^ Threonine. (D) 3D structural model of Mcoln1a channel. Green dot represents the central pore, and orange dots indicate the amino acid T519, which is mutated to I in *biluo* mutants. (E) Neutral red (NR) staining of 4-dpf *Tg*(*coro1a*:*mcoln1a*);*biluo* embryos indicates that the neutral red staining defect of microglia in *biluo* mutants can be rescued by ectopically expressing WT *mcoln1a* in microglia. White arrows represent microglia. Quantification data of neutral-red-positive microglia number in 4-dpf siblings, *biluo* mutants, and *biluo*;*Tg*(*coro1a*:*mcoln1a*) embryos. n(sibling) = 5 embryos, n(*biluo*) = 5 embryos, n(*biluo*;*Tg*(*coro1a*:*mcoln1a*)) = 5 embryos. ***p < 0.001, ANOVA. Error bars represent mean ± SD.

To identify the genetic lesion in *biluo* mutants, positional cloning was carried out. Bulk segregation analysis mapped the *biluo* mutation to chromosome 1, and subsequent fine-mapping analysis positioned the mutation on an 82-kb region containing four genes: *serhl*, *trappc5*, *mcoln1a*, and *si*:*Ch211-214C7*.*4* (Figure 2A). Sequencing of the coding regions of the four candidates revealed a point mutation in the *mcoln1a* gene, resulting in the substitution of a highly conserved Threonine (T) 519 with Isoleucine (I) (Figures 2B and 2C).

Mcoln1a is one of the two zebrafish counterparts of MCOLN1, a member of the mucolipin subfamily of transient receptor potential channels, which mediates the release of ions, such as Ca^2+^, from the lumen of LELs to cytosol (Dong et al., 2008, Dong et al., 2009, LaPlante et al., 2002, LaPlante et al., 2004, Luzio et al., 2007, Morgan et al., 2011). Previous studies have shown that multiple endocytic-related cellular processes are regulated by the Ca^2+^ released from lysosomal lumen to cytoplasm through the MCOLN1 channel (Dong et al., 2010, Luzio et al., 2007, Morgan et al., 2011). Based on recently solved Cryo-EM structure of mouse MCOLN1 (PDB: 5WPV) (Chen et al., 2017), we constructed a 3D homology model of zebrafish Mcoln1a and found the amino acid T519 is located in the sixth α-Helix of the transmembrane domain (Figure 2D). We therefore reasoned that the T to I mutation at residue 519 might be sufficient to disrupt the function of Mcoln1a channel, resulting in aberrant microglia phenotype in *biluo* mutants. To confirm this was indeed the case, we generated a *Tg*(*coro1a*:*mcoln1a*) transgenic line, in which WT *mcoln1a* is under the control of leukocyte-specific *coro1a* promoter (Li et al., 2012a). Whole-mount *in situ* hybridization and RT-PCR analysis confirmed that WT *mcoln1a* transgene was expressed specifically in myeloid precursors and microglia in *Tg*(*coro1a*:*mcoln1a*) transgenic fish (Figure S1). As anticipated, aberrant microglia phenotype was fully rescued in *biluo*;*Tg*(*coro1a*:*mcoln1a*) transgenic mutant fish (Figure 2E). Collectively, the genetic mapping and rescue results demonstrate that the microglia defect in *biluo* mutants is caused by this T to I mutation in the *mcoln1a* gene.

### The Aberrant Microglia Morphology in *biluo* Mutants Is Attributed to the Failure of Late Endosome and Lysosome Fusion

**Figure 3:**
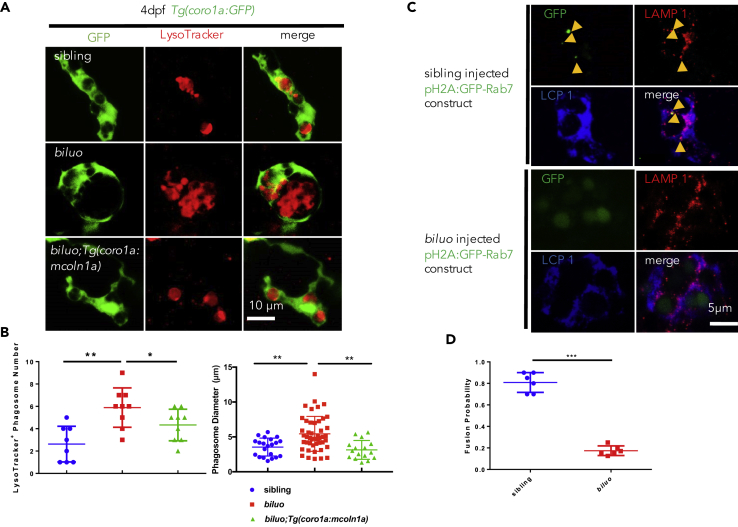
The Late Endosome and Lysosome Fusion Is Impaired in the *biluo* Microglia (A) Fluorescent imaging (green) and LysoTracker staining of 4-dpf sibling, *biluo*, and *Tg*(*coro1a*:*mcoln1a*);*biluo* embryos show that the accumulation of acid phagosomes in *biluo* mutant microglia (middle panel) and morphological defect of microglia in *biluo* mutants can be rescued by ectopically expressing WT *mcoln1a* in microglia (lower panel). (B) Quantification of the number and diameter of LysoTracker-positive phagosomes in the microglia in 4-dpf *Tg*(*coro1a*:*GFP*) siblings (blue dots), *Tg*(*coro1a*:*GFP*);*biluo* mutants (red squares), and *Tg*(*coro1a*:*mcoln1a*);*biluo* embryos (green triangles). n(sibling) = 8 microglia from 5 embryos; n(*biluo*) = 9 microglia from 5 embryos; n(*Tg*(*coro1a*:*mcoln1a*);*biluo*) = 9 microglia from 5 embryos. **p < 0.01, *p < 0.05, ANOVA. Error bars represent mean ± SD. (C) Anti-GFP (green), anti-LAMP1, and anti-Lcp1 (blue) triple antibody staining of WT embryos injected H2A:GFP-Rab7. Upper panels represent WT microglia (blue); late endosomes (green) are co-localized with the lysosomes. Lower panels represent mutant microglia (blue); late endosomes (green) are not co-localized with lysosomes. (D) Quantification of fusion probability of late endosomes and lysosomes in sibling microglia (blue dots) and mutant microglia (red squares). n(sibling) = 6 microglia from 4 embryos, n(*biluo*) = 6 microglia from 4 embryos. ***p < 0.001. Error bars represent mean ± SD.

Previous studies in the fibroblasts from human patients with ML-IV and in the fat bodies of *mcoln1*-deficient drosophila have shown that MCOLN1 plays a critical role in the fusion of phagosomes and lysosomes in the endocytosis traffic pathway (Chen et al., 1998, Wong et al., 2012). We therefore hypothesized that the accumulation of enlarged phagosomes in *biluo* mutant microglia was possibly due to the impairment of the late endosome and lysosome fusion. To support this hypothesis, we conducted LysoTracker DND-99 staining on 4-dpf *Tg(coro1a*:*GFP)*;*biluo* mutants and found that mutant microglia contained a significant increase of acid phagosomes in both number and diameter, which could be rescued by the reconstitution of WT Mcoln1a in microglia (Figures 3A and 3B). In contrast to *mcoln1ab* double-mutant zebrafish, MCOLN1-null mice, and *mcoln1*-deficient drosophila (Folkerth et al., 1995, Li et al., 2017, Wakabayashi et al., 2011), neither phagosome accumulation nor excessive cell death was detected in the neurons of *biluo* mutants during early development (Figures S2 and S3), suggesting a redundant role of *mcoln1b*. To confirm the enlarged phagosome phenotype in mutant microglia was indeed caused by the impaired late endosome and lysosome fusion, we injected pH2A:GFP-Rab7 DNA construct, in which the expression of late-endosome marker GFP-Rab7 fusion protein was under the control of the H2A promoter (Clark et al., 2011), into *biluo* mutants and siblings at one-cell stage. The embryos survived to 3 dpf and were stained with anti-GFP, anti-Lcp1, and anti-LAMP1 antibodies to monitor the formation of late endosomes (GFP) and lysosomes (LAMP1) and their subsequent fusion in microglia (Lcp1 positive) (Herbomel et al., 1999, Jin et al., 2012). As shown in Figure 3, the late endosomes (green, indicated by arrows) in the microglia of siblings were relatively small with condensed bright GFP signals and they were co-localized with the lysosomes (Figure 3C, upper panels), indicating successful fusion of the late endosomes and lysosomes. However, in the *biluo* mutant microglia, the late endosomes (green) were significantly enlarged with diffused faint GFP signals and they were clearly not co-localized with the lysosomes (Figure 3C, lower panels), indicating that late endosome and lysosome fusion is severely impaired in the mutant microglia. Quantification analysis showed that fusion between late endosomes and lysosomes was reduced for several-fold in *biluo* mutant microglia (Figure 3D). These data indicate that the aberrant morphology of microglia in *biluo* mutants is due to the failure of the late endosome and lysosome fusion, resulting in the accumulation of enlarged phagosomes in the cytosol. Because neutral red dyes are known to be enriched in the lysosomes through binding to lysosomal enzymes that are actively synthesized during phagocytosis (Chen et al., 1998), the failure of late endosome and lysosome fusion will likely interfere with the synthesis of the lysosomal enzymes, which leads to the reduction of neutral red staining in the *biluo* mutant microglia.

### The Impairment of Mcoln1a-Mediated Ca^2+^ Release Causes the Blockage of Late Endosome and Lysosome Fusion in *biluo* Mutant Microglia

**Figure 4:**
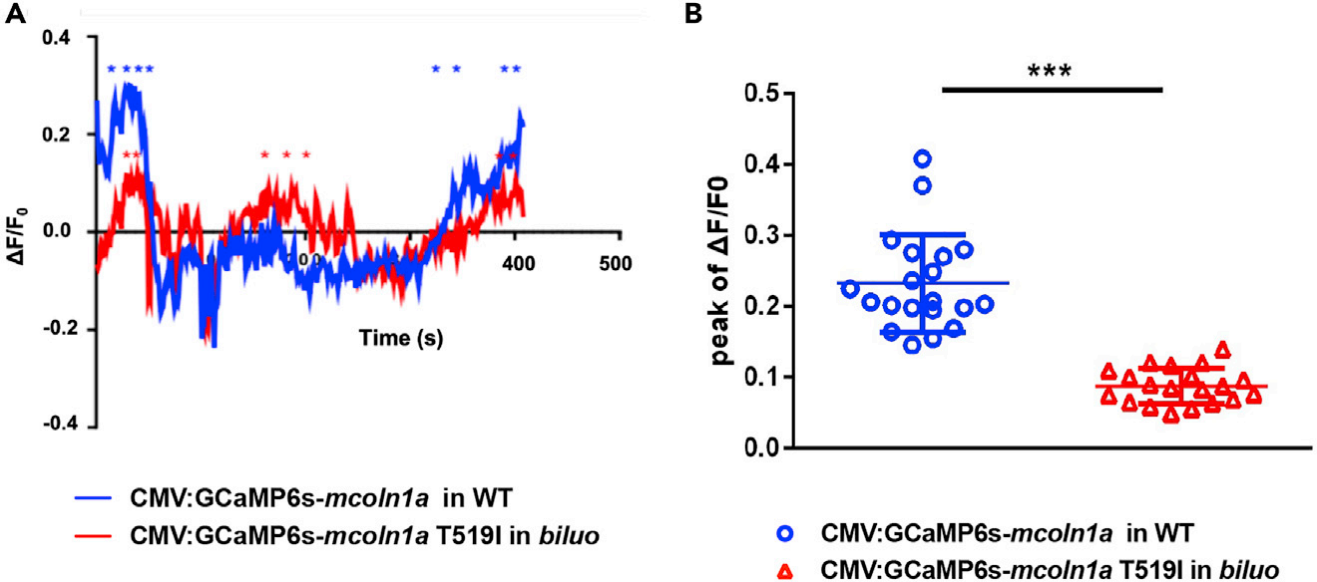
The T519 to I Mutation Reduces Mcoln1a-Mediated Ca^2+^ Efflux (A) Representative calcium efflux of WT (blue) and mutant Mcoln1a channel. Stars represent calcium peaks we quantified. (B) Quantification of the mean of the peak of ΔF/F0 (representing Trpml1a-mediated Ca^+^ efflux) in the macrophages in WT embryos injected with WT CMV:GCaMP6s-*mcoln1a* construct (blue dots) and the macrophages in *biluo* mutants injected with mutant CMV:GCaMP6s-*mcoln1a*T519I (red triangles). ΔF/F0 is calculated as (F-F0)/F0, where F0 is the baseline fluorescence signal. Error bars represent mean ± SD. n(WT late endosome) = 20 from 8 macrophages, n(*biluo* late endosome) = 20 form 7 macrophages.

As shown in the 3D structure model, the side chain of T519 faces the central ion permeation pore of the lower gate (Figure 2D), which directly contributes to ion release of the channel (Chen et al., 2017, Schmiege et al., 2017). We therefore speculated that, upon the replacement of Threonine by Isoleucine at residue 519, the hydrophobic side chain of Isoleucine might reduce the diameter of the central pore, thereby interfering with the calcium release of the channel. To test this hypothesis, we generated two constructions, CMV-GCaMP6s-*mcoln1a* and CMV-GCaMP6s-*mcoln1a*T519I, in which the calcium reporter GCaMP6s (Chen et al., 2013) was fused to the cytosolic N-terminal of WT Mcoln1a and mutant Mcoln1aT519I. The CMV-GCaMP6s-*mcoln1a* and CMV-GCaMP6s-*mcoln1a*T519I constructs were injected into one-cell stage WT and *biluo* embryos, respectively. The expression pattern and calcium efflux of WT Mcoln1a and Mcoln1aT519I mutant proteins were determined at around 2.5 dpf. Results showed that the GCaMP6s signals were co-localized with LysoTracker in the embryos injected with either WT Mcoln1a or mutant Mcoln1aT519I (Figure S4A), indicating that both WT GCaMP6s-Mcoln1a and mutant GCaMP6s-Mcoln1aT519I fusion proteins can properly target to the late endosomes and lysosomes. To monitor the function of WT and mutant Mcoln1a channels, we recorded the calcium efflux released from the late endosome in macrophages in the embryos injected with WT CMV-GCaMP6s-*mcoln1a* and mutant CMV-GCaMP6s-*mcoln1a*T519I constructs (Figure 4A). Quantification of the peak ΔF/F0 value (ΔF/F0 was calculated as (F-F0)/F0, where F0 is the baseline fluorescence signal) per late endosome showed that the calcium efflux mediated by Mcoln1aT519I mutant proteins was 2.5-fold lower than that of WT Mcoln1a (Figure 4B). To exclude the background effect of abnormal lysosomes/endosomes in the mutant microglia, we overexpressed WT GCAMP-Mcoln1a and mutant GCAMP-Mcol1aT519 in WT embryos and monitored the calcium efflux in WT macrophages. Results showed that the calcium efflux mediated by Mcoln1aT519I was also significantly reduced (Figure S4B). These data indicate that the blockade of the late endosome and lysosome fusion in *biluo* mutant microglia is likely attributed to the reduction of calcium released from the Mcoln1a channel.

### Aberrant Microglial Function Leads to Excessive Neuronal Activities in *Biluo* Mutants

**Figure 5:**
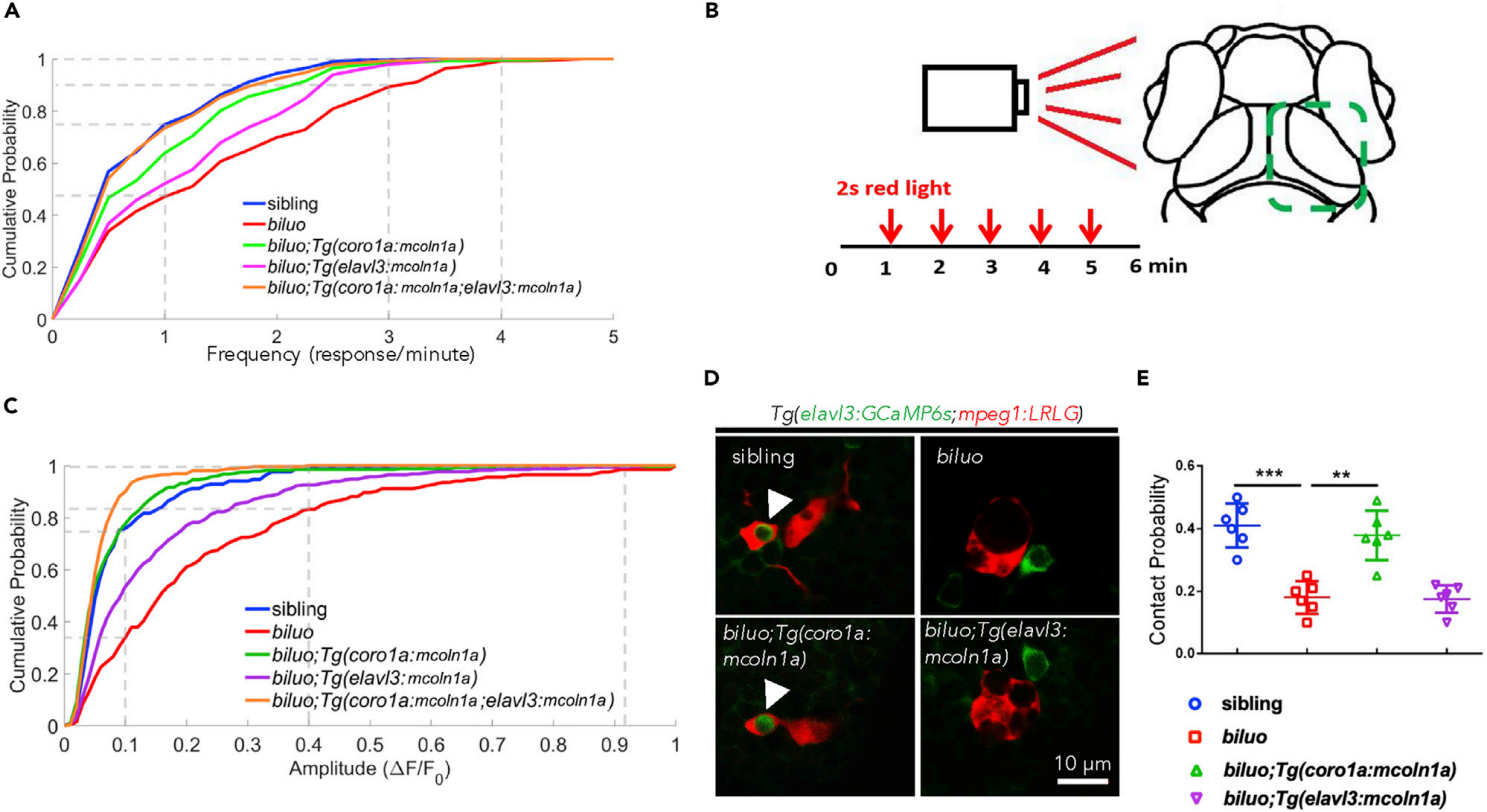
Optic Tectal Neurons in *biluo* Mutants Exhibit Excessive Spontaneous and Visual-Evoked Activities (A) Distribution of frequency of spontaneous responses of the tectal neurons in sibling, *biluo*, *biluo*;*Tg*(*coro1a*:*mcoln1a*), *biluo*;*Tg*(*elavl3*:*mcoln1a*), and *biluo*;*Tg*(*coro1a*:*mcoln1a*;*elavl3*:*mcoln1a*). n(sibling) = 214 cells in 4 embryos, n(*biluo*) = 166 cells in 4 embryos, n(*biluo*;*Tg*(*coro1a*:*mcoln1*)) = 165 cells in 4 embryos, n(*biluo*;*Tg*(*elavl3*:*mcoln1a*)) = 221 cells in 5 embryos and n(*biluo*;*Tg*(*coro1a*:*mcoln1a*;*elavl3*:*mcoln1a*)) = 225 cells in 4 embryos. P(sibling vs *biluo*) = 4.6 × 10^−13^, P(*biluo* vs *biluo*;*Tg*(c*oro1a*:mcoln1a)) = 9.4 × 10^−6^, P(*biluo* vs *biluo*;*Tg*(*elavl3*:*mcoln1a*)) = 0.0017, and P(*biluo* vs *biluo*;*Tg*(*coro1a*:*mcoln1a*;*elavl3*:*mcoln1a*)) = 5.7 × 10^−10^. (B) Schematic of recording visual-evoked responses of optic tectal neurons in 6-dpf embryos. Red light flashed was given to the left eye of the embryos at 1, 2, 3, 4, and 5 min, and the duration of each stimulus was 2 s. The neuronal responses of the right half tectum were recorded. (C) Distribution of average peak of ΔF/F0 in sibling, *biluo*, *biluo*;*Tg*(*coro1a*:*mcoln1a*), *biluo*;*Tg*(*elavl3*:*mcoln1a*), and *biluo*;*Tg*(*coro1a*:*mcoln1*;*elavl3*:*mcoln1*). n(sibling) = 203 cells in 4 embryos, n(*biluo*) = 263 cells in 4 embryos, n (*biluo*;*Tg*(*coro1a*:*mcoln1a*)) = 305 cells in 4 embryos, n(*biluo*;*Tg*(*elavl3*:*mcoln1a*)) = 364 cells in 5 embryos, n(*biluo*;*Tg*(*coro1a*:*mcoln1a*;*elavl3*:*mcoln1a*)) = 280 cells in 4 embryos. P(sibling vs *biluo*) = 4.9 × 10^−17^, P(*biluo* vs *biluo*;*Tg*(*coro1a*:*mcoln1a*)) = 4.7 × 10^−22^, P(*biluo* vs *biluo*;*Tg*(*elavl3*:*mcoln1a*)) = 4.2 × 10^−6^, P(*biluo* vs *biluo*;*Tg*(*coro1a*:*mcoln1a*;*elavl3*:*mcoln1a*)) = 3.2 × 10^−34^, and P(*biluo*;*Tg*(*coro1a*:*mcoln1a*) vs *biluo*;*Tg*(*elavl3*:*mcoln1a*)) = 2.1 × 10^−11^. (D) Representative images of neuron (green)-microglia contact in 5-dpf sibling, *biluo*, *biluo*;*Tg*(*coro1a*:*mcoln1a*), and *biluo*;*Tg*(*elavl3*:*mcoln1a*) embryos. (E) Quantification of contact probability between microglia and optic tectal neurons. Contact duration longer than 24 s is recognized as a functional microglia-neuron contact. n(sibling) = 9 embryos, n(*biluo*) = 10 embryos, n (*biluo*;*Tg*(*coro1a*:*mcoln1a*)) = 9 embryos, n (*biluo*;*Tg*(*elavl3*:*mcoln1a*)) = 8 embryos. ***p < 0.0001, **p < 0.001, ANOVA. Error bars represent mean ± SD.

It is well known that mutations in the *MCOLN1* gene in humans cause ML-IV disorder, a neurodegenerative and lysosomal storage disorder with various neuronal symptoms, including psychomotor retardation and vision impairment (Bargal et al., 2000, Bassi et al., 2000, Wakabayashi et al., 2011). Animal model studies in mice, fish, and fly have shown that these various neuronal symptoms are believed to be caused, at least in part, by the intrinsic defects in the neurons (Li et al., 2017, Micsenyi et al., 2009, Venkatachalam et al., 2008, Venugopal et al., 2007). Yet, the role of microglia in ML-IV disorder remains elusive, despite the fact that microglia exhibit abnormal morphology in human patients with ML-IV and *MCOLN1*-deficient mice (Folkerth et al., 1995). Given the fact that *biluo* mutant microglia display an aberrant morphology similar to those in human patients with ML-IV, the mutant fish provide a useful platform to address this question. Because *biluo* is a hypomorphic mutant and neuronal behavior study is less established in adult zebrafish, we decided to examine the spontaneous and visual-evoked neuronal activities, both of which have been shown to be modulated by microglia at early developmental stage (Li et al., 2012b), in *biluo* mutants. We crossed *Tg(elavl3*:*GCaMP6s)* line, in which the calcium indicator *GCaMP6s* is selectively expressed in the neurons, with *biluo* mutants as well as three different mutant transgenic lines, *biluo*;*Tg*(*coro1a*:*mcoln1a*), *biluo*;*Tg*(*elavl3*:*mcoln1a*), and *biluo*;*Tg*(*coro1a*:*mcoln1a*;*elavl3*:*mcoln1a*), ectopically expressing WT *mcoln1a* in microglia and neurons alone or together (Figures S1 and S5). At 6 dpf, the neuronal activities in the optic tectum, where microglia predominantly reside in developing zebrafish brain (Xu et al., 2015, Xu et al., 2016), were analyzed under both non-stimulated and visual-evoked conditions. For spontaneous neuronal activity, we calculated the frequency of calcium events per neuron and plotted the cumulative curves for the distribution of neuronal frequency (Figure 5A). In control siblings, more than 70% of the neurons showed one event or less per minute and none of the neurons exhibited three to four events per minute (Figure 5A). Intriguingly, in *biluo* mutants, 10% of the tectal neurons had three to four events per minute and the neurons with the frequency less than one event per minute were reduced to 50% (Figure 5A), indicating that the optic tectal neurons in *biluo* mutants display excessive spontaneous activities. Remarkably, these excessive neuronal activities were partially rescued by ectopically expressing WT *mcoln1a* in either neurons or microglia and were fully rescued by expressing WT *mcoln1a* in the neurons and microglia together (Figure 5A).

We next explored the visual-evoked activities of the tectal neurons in *biluo* mutants by recording the neuronal activities on the contralateral optic tectum after stimulating the larva with one-side red light flashes lasting for 2 s for five times with 1-min intervals (Figure 5B). To quantify the visual-evoked responses of tectal neurons, we averaged the five-time responses (5 s after stimulus) for each neuron by averaging the peak of ΔF/F0 (ΔF/F0 was calculated as (F-F0)/F0, where F0 is average fluorescence during 5 s before stimulus). The cumulative curves for the distribution of the peaks of tectal neuron's responses in siblings, *biluo*, *biluo*;*Tg*(*coro1a*:*mcoln1a*), *biluo*;*Tg*(*elavl3*:*mcoln1a*) and *biluo*;*Tg*(*coro1a*:*mcoln1a*;*elavl3*:*mcoln1a*) mutants, were plotted (Figure 5C). Results showed that, similar to the spontaneous neuronal activity, the visual-evoked tectal responses were also significantly higher in *biluo* mutants and these excessive neuronal responses could be rescued by ectopically expressing WT *mcoln1a* in microglia. Taken together, these data indicate that, in addition to the intrinsic neuronal impairment, aberrant microglial function also contributes to the dysregulation of neuronal activities in *mcoln1a*-deficient fish.

Previous study by Li et al. have reported that microglia preferentially interact with highly activated neurons by wrapping around their soma into a bulbous structure, leading to the reduction of the activities of these neurons (Li et al., 2012b). We therefore hypothesized that the excessive neuronal activities in *biluo* mutants might be due to the impairment of the neuron-microglia interaction. Based on previous study (Li et al., 2012b), a functional neuron-microglia contact will be achieved when microglia form a bulbous structure on a neuron soma for more than 24 s. We therefore compared the contact probability between the tectal neurons and microglia in the optic tectum of siblings, *biluo*, *biluo*;*Tg*(*coro1a*:*mcoln1a*), and *biluo*;*Tg*(*elavl3*:*mcoln1a*) mutant fish. Results showed that the probability of functional neuron-microglia contacts in *biluo* mutants was about a half of that in siblings (Figures 5D and 5E). This impaired contact probability in *biluo* mutants was restored by ectopically expressing WT Mcoln1a in microglia but not in neurons (Figures 5D and 5E). Collectively, these results indicate that the excessive neuronal activities of the tectal neurons in *biluo* mutants is attributed, at least in part, to the impairment of direct interaction between neurons and microglia.

## Discussion

In this study, we reported the identification and characterization of a zebrafish mutant *biluo*, which harbors a hypomorphic mutation in the *mcoln1a* gene, one of the two zebrafish homologs of mammalian *MCOLN1*. Our study showed that loss of Mcoln1a function impairs the microglia-neuron interaction, resulting in excessive neuronal activities.

It is well known that mutations in the *MCOLN1* gene in human cause type IV mucolipidosis (ML-IV), a neurodegenerative and lysosomal storage disorder that displays various neurological symptoms (Bargal et al., 2001, Bassi et al., 2000, Wakabayashi et al., 2011). The hallmark of ML-IV disease is that the patients often suffer from progressive psychomotor retardation, especially their movement and coordination, in an age-dependent manner (Bach, 2001). However, we did not observe excessive neuronal cell death, movement, and coordination defects in *biluo* mutants (data not shown). This is likely attributed to the hypomorphic T to I mutation of Mcoln1a as well as the functional compensation of Mcoln1b, which shares 60% similarity in protein sequence with Mcoln1a. Indeed, excessive neuronal cell death was detected in the complete loss of *mconl1a* function mutant fish and *mconl1ab* double-deficient fish (Li et al., 2017). Further in-depth study will be required to clarify whether complete loss of Mcoln1 function in zebrafish would lead to movement and coordination defects. Nevertheless, as missense mutations in the *MCOLN1* gene have been found to cause mild symptoms in human patients (Bargal et al., 2001, Reis et al., 1993), we believe that the *biluo* mutants may represent a mild ML-IV disease model for the mechanistic study of the disease and drug screening.

Although *biluo* mutants lack movement and coordination phenotypes, they exhibit excessive neuronal activities under spontaneous and visual-evoked conditions. These aberrant neuronal activities appear to be caused by the intrinsic defect of neurons as well as the extrinsic contribution from the microglia. Previous studies in the *mcoln1*-deficient drosophila have shown that the movement and coordination defects in the mutant fly are caused by excessive neuronal cell death (Venkatachalam et al., 2008). Intriguingly, no obvious abnormal neural cell death is observed in *biluo* mutants, suggesting that the abnormal neuronal activities in *biluo* mutants is likely not caused by excessive neuronal cell death. It is known that the impairment of endosomal fusion pathway could interfere with the neurotransmitter recycling process (Rizzoli, 2014). The partial loss of Mcoln1a function could reduce the degradation of neurotransmitters in neurons due to the impaired fusion of transmitter vesicles with lysosomes. As a consequence, more neurotransmitters will be released to the post-synaptic membrane, resulting in a stronger spontaneous and visual-evoked neuronal response. Likewise, as microglia have also been implicated in the removal of neurotransmitters via phagocytosis (Shaked et al., 2005), the excessive spontaneous and visual-evoked neuronal activities in *biluo* mutants could contribute to the impairment of removing neurotransmitters by microglia. Indeed, when we injected bacteria into the brain of 4-dpf *biluo* mutant embryos, we found that the ability of engulfing the bacteria by the mutant microglia was significantly reduced (Figure S6). This observation supports the hypothesis that the excessive neuronal response in *biluo* mutants could be attributed to the impairment of neurotransmitter recycling.

Finally, previous studies have reported that aberrant microglia activity contribute to the onset and the progression of a number of neurodegenerative diseases, including Alzheimer and Parkinson diseases, Amyotrophic lateral sclerosis, and prion disease, presumably by releasing pro-inflammatory and neurotoxic factors (Block et al., 2007, Kim et al., 2015, Qin et al., 2004, Takeuchi et al., 2006, Thompson and Tsirka, 2017). Interestingly, our study shows that the aberrant microglial function caused by *mcoln1a* deficiency could lead to abnormal excessive neuronal activities. This finding suggests that microglia-neuron interaction may play a role in the development and progression of ML-IV disease as well as some other neurological disorders. One example is Fragile X syndrome (FXS), a type of Autism spectrum disorder caused by developmental defects in brain connectivity (Geschwind and Levitt, 2007). The FXS mouse model shows abnormal high synchrony in the firing of cortical neurons (Goncalves et al., 2013). It will be of great interest to explore the role of microglia in the FXS mouse model.

### Limitations of the Study

This study has demonstrated that loss of Mcoln1a function in zebrafish impairs microglia-neuron interaction, which leads to excessive neuronal activities in the mutant animals. However, whether dysregulation of microglial function contributes to the development of type IV mucolipidosis needs to be further investigated in human patients.

## Methods

All methods can be found in the accompanying Transparent Methods supplemental file.

## Supplemental Information

Document S1. Transparent Methods and Figures S1–S6
