# Supplementary material for: Dysregulation of Microglial Function Contributes to Neuronal Impairment in Mcoln1a-Deficient Zebrafish
Source: iScience. 2019 Mar 2;13:391–401. doi: 10.1016/j.isci.2019.02.031 (PMC6426713; doi:10.1016/j.isci.2019.02.031)
Supplement: Document S1. Transparent Methods and Figures S1–S6 [file mmc1.pdf]

ISCI, Volume *13*

Supplemental Information

Dysregulation of Microglial Function Contributes to Neuronal Impairment in Mcoln1a-Deficient Zebrafish

Wan Jin, Yimei Dai, Funing Li, Lu Zhu, Zhibin Huang, Wei Liu, Jianchao Li, Mingjie Zhang, Jiulin Du, Wenqing Zhang, and Zilong Wen

SUPPLEMENTAL FIGURES AND LEGENDS

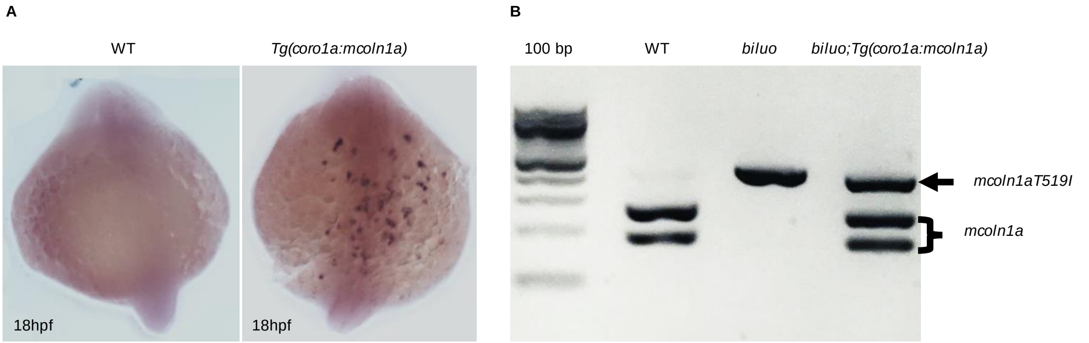

Figure S1 related to Figure 2. **The Expression of WT *mcoln1a* in *Tg(coro1a:mcoln1a)* Transgenic and *biluo;Tg(coro1a:mcoln1a)* Mutant Transgenic Embryos.**

(A) *In-situ* hybridization of *mcoln1a* in myeloid precursors in *Tg(coro1a:mcoln1a)* transgenic and control WT embryos at 18 hpf.

(B) The *mcoln1a* transcripts in microglia are detected by RT-PCR analysis of the microglia isolated from the brains of 3 dpf *Tg(mpeg1:LRLG)*, *biluo;Tg(mpeg1:LRLG)* and *biluo;Tg(coro1a:mcoln1a;mpeg1:LRLG)* embryos. The PCR product of WT *mcoln1a* can be cleaved into two smaller fragments (~200 bp) by MspI, whereas the PCR product of mutant *mcoln1a* is resistant to MspI digestion.

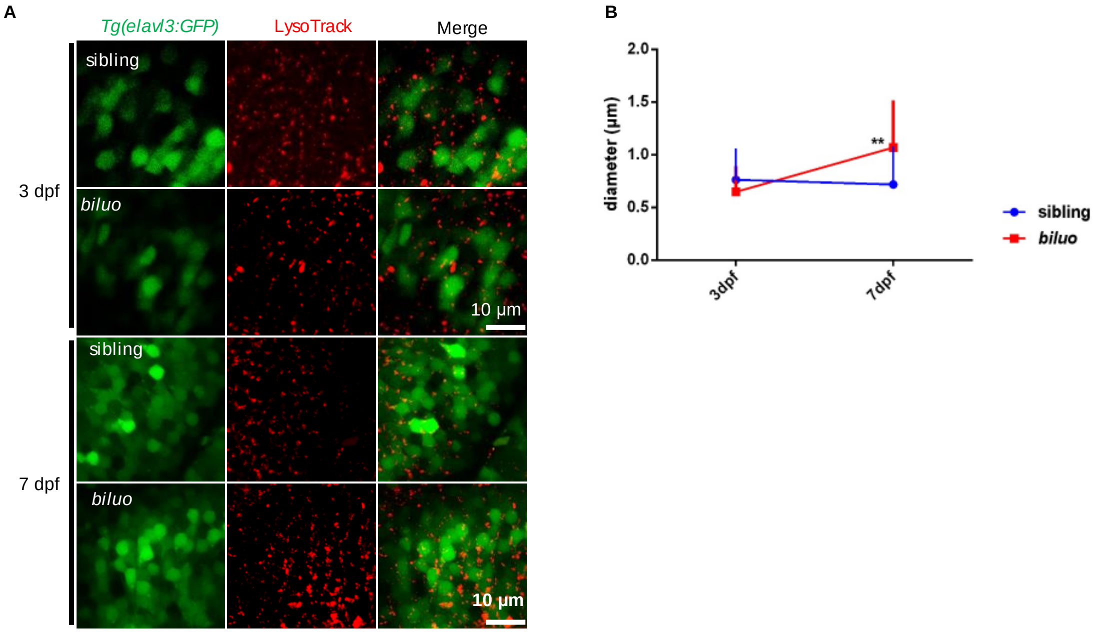

Figure S2 related to Figure 3. **Lysosomal Accumulation in the Neurons of *biluo* Mutants.**

(A) LysoTracker staining (red) of neurons (green) in both *Tg(elavl3:GFP)* and *biluo;Tg(elavl3:GFP)* at 3 dpf (upper panels) and 7 dpf (lower panels).

(B) Quantification data of the diameter of LysoTracker positive lysosomal accumulation. n(4 dpf sibling)=5 embryos, n(4 dpf *biluo*)=5 embryos, n(7 dpf sibling)=4 embryos, n(7 dpf *biluo*)=5 embryos. **P<0.01 Error bars represent mean ± SD.

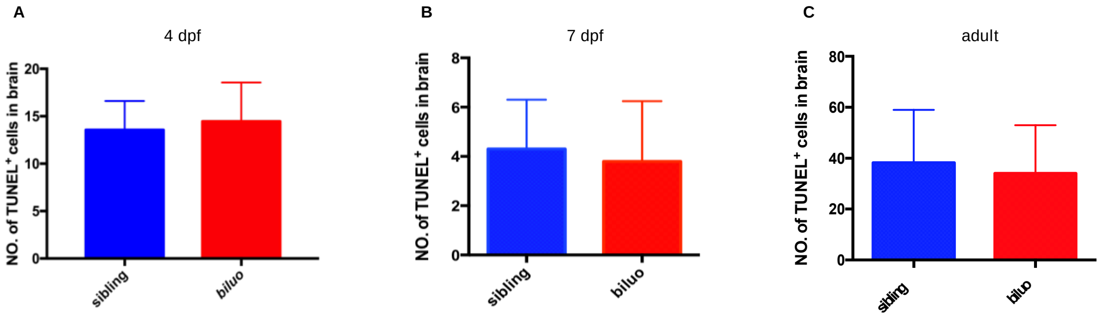

Figure S3 related to Figure 3. **Terminal Deoxynucleotidyl Transferase dUTP Nick End Labeling (TUNEL) Staining in Embryonic and Adult Brains of *biluo* Mutants.**

(A) Quantification data of the number of TUNEL positive cells in 4 dpf mid-brain in siblings (blue) and *biluo* mutants (red). n(sibling)=9 embryos, n(*biluo*)=11 embryos, p=0.765. Error bars represent mean ± SD.

(B) Quantification data of the number of TUNEL positive cells in 7 dpf mid-brain in siblings (blue) and *biluo* mutants (red). n(sibling)=10 embryos, n(biluo)=10 embryos, p=0.623. Error bars represent mean ± SD.

(C) Quantification data of the number of TUNEL positive cells in adult brain in siblings (blue) and *biluo* mutants (red). n(sibling)=4 fish, n(*biluo*)=4 fish, p=0.7726. Error bars represent mean ± SD.

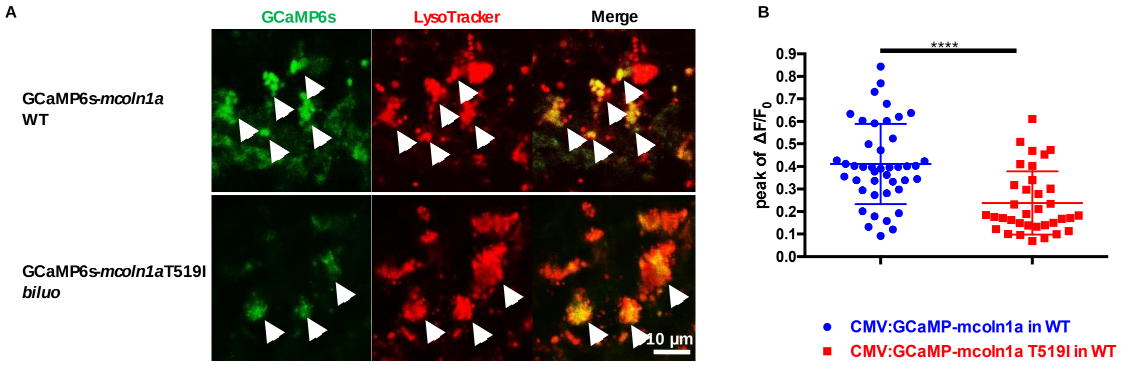

Figure S4 related to Figure 4. **Co-localization of WT and Mutant GCaMP6s-Mcoln1a with LysoTracker.**

(A) Anti-GFP and LysoTracker double staining shows co-localization of LysoTracker with WT (GCaMP6s-*mcoln1a*) and mutant (GCaMP6s-*mcoln1a* T519I) proteins in WT and *biluo* embryos injected with the WT GCaMP6s-*mcoln1a* and mutant GCaMP6s-*mcoln1a*T519I constructs respectively. White arrows indicate the co-localization.

(B) Quantification of the mean of the peak of ΔF/F0 (representing Trpml1a-mediated Ca^+ efflux) in the macrophages in WT embryos injected with WT CMV:GCaMP6s-*trpml1a* construct (blue dots) and mutant CMV:GCaMP6s-*trpml1a* T519I (red square). ΔF/F0 is calculated as (F-F0)/F0, where F0 is the baseline fluorescence signal. **** P < 0.0001, Error bars represent mean±SD. n(WT late endosome)=43, n(*biluo* late endosome)=35

3 dpf, *mcoln1a* expression, FITC

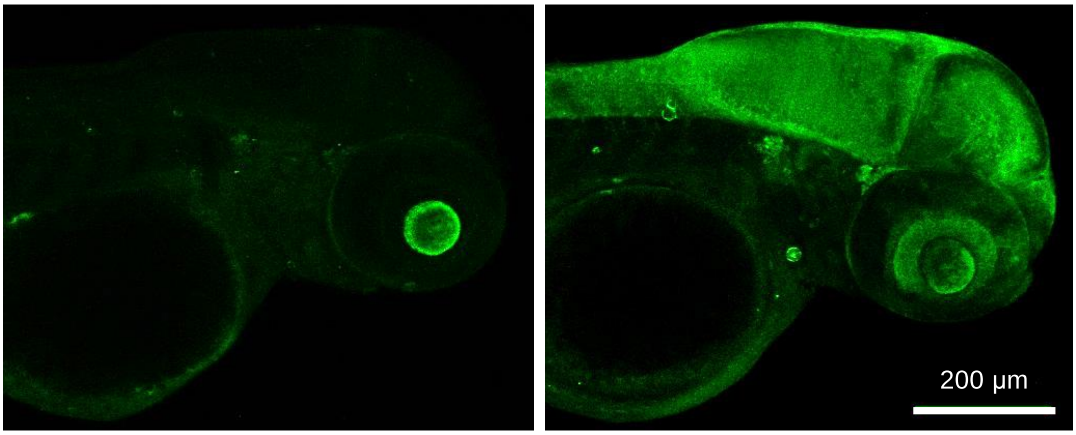

Figure S5 related to Figure 5. **The Expression of *mcoln1a* in *Tg(elval3:mcoln1a)*.** *In-situ* hybridization indicates *mcoln1a* expression in *Tg(elavl3:mcoln1a)* and WT embryos at 3 dpf.

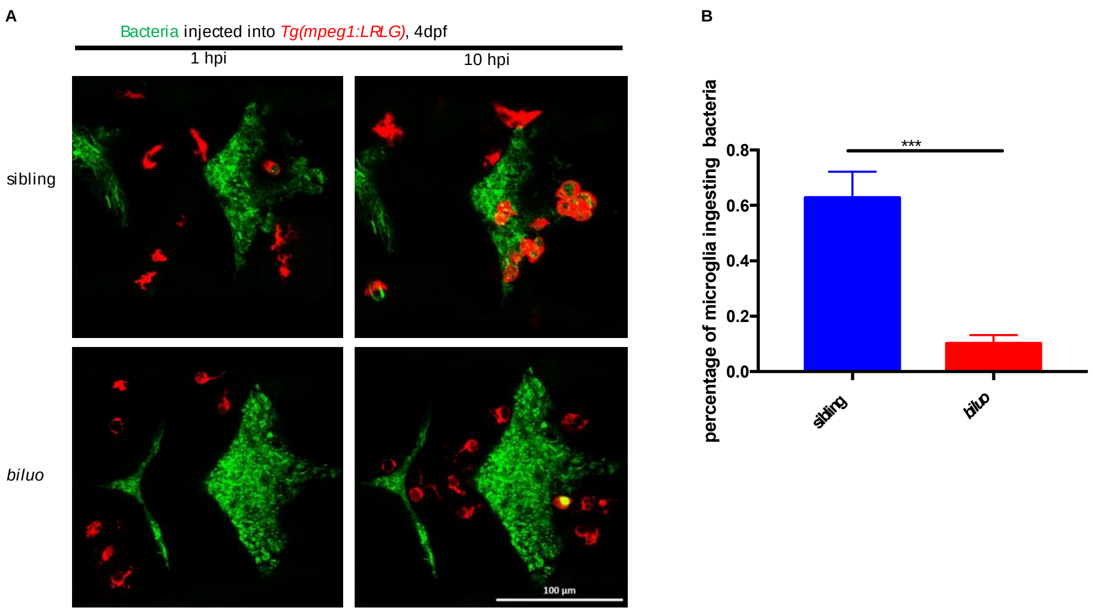

Figure S6 related to Figure 5. **Microglia in *biluo* Mutants Have Decreased Engulfment Ability.**

(A) GFP bacteria was injected into the brain of sibling (upper panels) and *biluo* mutants (lower panels) at 4 dpf and images were taken at 1 hpi (left panels) and 10 hpi (right panels).

(B) Quantification of the percentage of microglia engulfing green bacteria at 10 hpi. n(sibling)=4, n(*biluo*)=4. ***P<0.001, t-test. Error bars represent mean ± SD.

TRANSPARENT METHODS

Zebrafish

Zebrafish were raised and maintained as described (Westerfield). AB, WIK strain, *biluo*^{hkz12}, *Tg(coro1a:GFP)* (Li et al., 2012a), *Tg(hsp70:mCherry-T2a-CreERT2)#12* (Hans et al., 2011), *Tg(mpeg1:loxP-DsRedx-loxP-GFP)hkz15Tg* (Xu et al., 2015), *Tg(elavl3:GCaMP6s) (Cong et al., 2017)*, *Tg(coro1a:mcoln1a)hkz20Tg* and *Tg(elavl3:mcoln1a)hkz21Tg* were used in this study. All experiments were conducted with approval of the Animal Ethics Committee of the Hong Kong University of Science and Technology.

Ethylnitrosourea (ENU) Mutagenesis and Neutral Red Staining

ENU (Sigma, USA) mutagenesis was carried out as described (Mullins et al., 1994; Solnica-Krezel et al., 1994). Neutral red staining was performed as reported (Herbomel et al., 2001).

Positional Cloning

Positional cloning was performed as described previously (Bahary et al., 2004). Bulk two simple sequence length polymorphism markers (SSLPs) markers zC214C7I and 211668r. Sequencing of candidate genes within the region revealed a C to T mutation in the *mcoln1a* gene.

Generation of Transgenic Lines

The coding regions of the *mcoln1a* gene was placed under the control of the *elavl3* and *coro1*a promoters and inserted into the pTol2 vector. The generation of transgenic fish lines were performed as described (Kawakami et al., 2000).

Whole-Mount *in situ* Hybridization (WISH)

Antisense DIG labeled RNA probes of *mcoln1a* was synthesized *in vitro*. WISH was carried out according to the standard protocol (Westerfield).

Whole-Mount Antibody Staining

Whole-mount antibody staining was performed as described (Barresi et al., 2000; Jin

et al., 2009). The primary antibodies used in this study are: Anti-GFP antibody (ab6658, Abcam; 1:800 dilution), Anti-LAMP1 antibody (ab24170, Abcam; 1:800 dilution), Anti-Lcp1 antibody (1:400 dilution) (Jin et al., 2009) and Anti-Mcoln1a antibody (1:400 dilution). The secondary antibodies used are: Alexa 488-anti-goat antibody (A11055 Invitrogen), Alexa 555-anti-rabbit antibody (A31572 Invitrogen) and Alexa 647-anti-rabbit antibody (A31573 Invitrogen). Images were captured by Leica SP8 confocal microscope.

Terminal Deoxynucleotidyl Transferase dUTP Nick End Labeling (TUNEL) Staining

TUNEL staining was performed using the *In Situ* Cell Death Detection Kit TMR red (Roche) according to the manufacturer's instructions.

LysoTracker Staining

Embryos were maintained in egg water containing PTU (1-phenyl 2-thiourea) and incubated with LysoTracker™ Red DND-99 (Invitrogen™) at 50 nM for 10 minutes. After washing with egg water for three times, the embryos were imaged by Leica SP8 confocal microscope. Microglia in the optic tectum of brain were imaged, whereas peripheral macrophages in the CHT region were imaged.

Calcium Release Assay

We injected CMV:GCaMP6s-*mcoln1a* or CMV:GCaMP6s-*mcoln1a*T519I into WT or *biluo* mutant embryos. Around 2 dpf, before microglia precursors entering into brain, we performed *in vivo* live imaging to record calcium release of Mcoln1a channel in peripheral macrophages in the CHT region. Individual late endosomes or lysosomes were manually segmented on the average image after registration by MultiStackReg plugin in ImageJ. De-trend and noise reduction were performed by a self-written MATLAB program. Calcium events were detected as peaks by a self-written MATLAB program. For each late endosome or lysosome, we averaged the amplitude of calcium peak as its amplitude of calcium release during the recording time.

Single Microglia Collection and Genotyping

In this experiment, three types of 3 dpf embryos, *Tg(mpeg1:LRLG), biluo;Tg(mpeg1-LRLG)* and *biluo;Tg(mpeg1-LRLG;coro1a:mcoln1a),* were used. In each group, the brains of 15 embryos with strong red fluorescence were dissected and dissociated by Dispase (Roche) to single cell solution. Then single mpeg1$^+$ cells (microglia) with intact morphology under fluorescent microscope was picked manually and immediately suspended in lysis buffer. Two single cells were pulled together to generate cDNA libraries using Smart-seq2 method (Picelli et al., 2014). Primers used for genotyping are: FP-CTGTTGCTGTGTGGCGGTGATCT, RP-GTTGACAAGGAGGACGTCCTCA. WT *mcoln1a* but not mutant *mcoln1aT519I* PCR products can be digested by MspI into two 200 bp fragments.

In vivo **Confocal Calcium Imaging**

Larval zebrafish (6 dpf) were embedded in 1.5% low-melting agarose after paralysis by 0.1% α-bungarotoxin. Calcium images were taken by an Olympus Fluoview 1000 upright confocal microscope (Tokyo, Japan) with a 40× water-immersion objective (NA = 0.80). Frames with an approximate 150×150 μm^2 field of view of one-side optic tectum (about 350 ×350 pixel^2 and 0.397 ×0.397 μm^2 per pixel) were acquired at about 1.2 Hz. Individual neurons were manually segmented on the average image after registration by MultiStackReg plugin in ImageJ. De-trend and noise reduction were performed by a self-written MATLAB program. Fluorescence lower than 10 % (threshold) was reassigned zero. Calcium events were detected as peaks by a self-written MATLAB program. For visual responses, a true positive response was identified if the maximal fluorescence after stimulation is larger than ± 2 SD of the baseline.

Visual Stimulation

Light flashes were generated by a red LED to avoid the interference of spectrum to cesium imaging. The LED and imaging setup were coupled by Master 8. Visual stimuli included 5 trials and each flash lasted for 2 second with 60 s interval. Visual stimuli were shed on one-side of eye and calcium images were taken on the contralateral optic tectum according to retinotopic maps.

Statistical Analysis

F-test was performed to test two sample variances. According to the results of F-test, two-sample T-test was performed assuming equal or unequal variances. Statistical analysis was performed by Data Analysis tool of Excel software (Microsoft). Two-tailed P values were used in all T tests. To make multi-group comparison, one-way ANOVA was performed followed by Tukey's multiple comparisons test. ANOVA analysis was performed by GraphPad software. In calcium imaging experiment, Kolmogorov-Smirnov test was applied in Matlab.

SUPPLEMENTAL REFERENCES

Bahary, N., Davidson, A., Ransom, D., Shepard, J., Stern, H., Trede, N., Zhou, Y., Barut, B., and Zon, L.I. (2004). The Zon laboratory guide to positional cloning in zebrafish. Methods in cell biology *77*, 305-329.

Barresi, M.J., Stickney, H.L., and Devoto, S.H. (2000). The zebrafish slow-muscle-omitted gene product is required for Hedgehog signal transduction and the development of slow muscle identity. Development *127*, 2189-2199.

Cong, L., Wang, Z., Chai, Y., Hang, W., Shang, C., Yang, W., Bai, L., Du, J., Wang, K., and Wen, Q. (2017). Rapid whole brain imaging of neural activity in freely behaving larval zebrafish (Danio rerio). eLife *6*.

Hans, S., Freudenreich, D., Geffarth, M., Kaslin, J., Machate, A., and Brand, M. (2011). Generation of a non-leaky heat shock-inducible Cre line for conditional Cre/lox strategies in zebrafish. Developmental dynamics: an official publication of the American Association of Anatomists *240*, 108-115.

Jin, H., Sood, R., Xu, J., Zhen, F., English, M.A., Liu, P.P., and Wen, Z. (2009). Definitive hematopoietic stem/progenitor cells manifest distinct differentiation output in the zebrafish VDA and PBI. Development *136*, 647-654.

Kawakami, K., Shima, A., and Kawakami, N. (2000). Identification of a functional transposase of the Tol2 element, an Ac-like element from the Japanese medaka fish, and its transposition in the zebrafish germ lineage. Proc Natl Acad Sci U S A *97*, 11403-11408.

Li, L., Yan, B., Shi, Y.Q., Zhang, W.Q., and Wen, Z.L. (2012a). Live imaging reveals differing roles of macrophages and neutrophils during zebrafish tail fin regeneration. The Journal of biological chemistry *287*, 25353-25360.

Mullins, M.C., Hammerschmidt, M., Haffter, P., and Nusslein-Volhard, C. (1994). Large-scale mutagenesis in the zebrafish: in search of genes controlling development in a vertebrate. Current biology: CB *4*, 189-202.

Picelli S, Faridani O R, Björklund A K, et al. Full-length RNA-seq from single cells using Smart-seq2.[J]. Nature Protocols, 2014, 9(1):171-181.

Solnica-Krezel, L., Schier, A.F., and Driever, W. (1994). Efficient recovery of ENU-induced mutations from the zebrafish germline. Genetics *136*, 1401-1420.

Westerfield, M. *The zebrafish book: A Guide for the Laboratory Use of Zebrafish (Danio rerio) 3rd ed* (Eugene, OR: University of Oregon Press, 1995).

Xu, J., Zhu, L., He, S., Wu, Y., Jin, W., Yu, T., Qu, J.Y., and Wen, Z. (2015). Temporal-Spatial Resolution Fate Mapping Reveals Distinct Origins for Embryonic and Adult Microglia in Zebrafish. Developmental cell *34*, 632-641.
